# Unveiling a Biomarker Signature of Meningioma: The Need for a Panel of Genomic, Epigenetic, Proteomic, and RNA Biomarkers to Advance Diagnosis and Prognosis

**DOI:** 10.3390/cancers15225339

**Published:** 2023-11-09

**Authors:** Reem Halabi, Fatima Dakroub, Mohammad Z. Haider, Stuti Patel, Nayef A. Amhaz, Mohammad A. Reslan, Ali H. Eid, Yehia Mechref, Nadine Darwiche, Firas Kobeissy, Ibrahim Omeis, Abdullah A. Shaito

**Affiliations:** 1 Department of Biological and Chemical Sciences, Lebanese International University, Beirut 1105, Lebanon; reemhalabi5@gmail.com; 2 Department of Experimental Pathology, Microbiology and Immunology and Center for Infectious Diseases Research, Faculty of Medicine, American University of Beirut, Beirut 1107, Lebanon; fd31@aub.edu.lb; 3 Department of Basic Medical Sciences, College of Medicine, QU Health, Qatar University, Doha P.O. Box 2713, Qatar; mh1704315@student.qu.edu.qa (M.Z.H.); ali.eid@qu.edu.qa (A.H.E.); 4 Department of Biology, University of Florida, Gainesville, FL 32601, USA; patel.stuti@ufl.edu (S.P.); nayefamhaz@ufl.edu (N.A.A.); 5 Department of Biochemistry and Molecular Genetics, American University of Beirut, Beirut 1107, Lebanon; moha.reslan@gmail.com (M.A.R.); nd03@aub.edu.lb (N.D.); firasko@gmail.com (F.K.); 6 Department of Chemistry and Biochemistry, Texas Tech University, Lubbock, TX 79409, USA; yehia.mechref@ttu.edu; 7 Department of Neurobiology, Center for Neurotrauma, Multiomics & Biomarkers (CNMB), Morehouse School of Medicine, Atlanta, GA 30310, USA; 8 Hammoud Hospital University Medical Center, Saida 652, Lebanon; 9 Division of Neurosurgery, Penn Medicine, Lancaster General Health, Lancaster, PA 17601, USA; 10 Biomedical Research Center, College of Medicine, and Department of Biomedical Sciences at College of Health Sciences, Qatar University, Doha P.O. Box 2713, Qatar

**Keywords:** meningioma, *NF2* mutations, biomarker, miRNA, proteomics

## Abstract

**Simple Summary:**

MRI and histological assessment remain the gold standard for meningioma diagnosis. Currently, WHO grading of meningiomas mainly depends on histologic and morphological markers and two molecular markers. WHO grading can reliably diagnose meningiomas in most cases. However, it was not as dependable in predicting prognosis, especially time to recurrence of Grade 1 and 2 meningiomas. This warrants the integration of new biomarkers into the current WHO grading system of meningiomas. Future meningioma biomarkers need to utilize an array of molecular technologies for biomarkers discovery, including genomic, epigenetic, proteomis, metabolomic, and RNA biomarkers, as well as a panel format to complement the existing WHO grading. The majority of candidate meningioma molecular biomarkers are still experimental and need to undergo testing in clinical trials, but their application in meningioma diagnosis will be necessary to guide future targeted therapies of meningiomas.

**Abstract:**

Meningiomas are the most prevalent primary intracranial tumors. The majority are benign but can undergo dedifferentiation into advanced grades classified by World Health Organization (WHO) into Grades 1 to 3. Meningiomas’ tremendous variability in tumor behavior and slow growth rates complicate their diagnosis and treatment. A deeper comprehension of the molecular pathways and cellular microenvironment factors implicated in meningioma survival and pathology is needed. This review summarizes the known genetic and epigenetic aberrations involved in meningiomas, with a focus on neurofibromatosis type 2 (*NF2*) and non-*NF2* mutations. Novel potential biomarkers for meningioma diagnosis and prognosis are also discussed, including epigenetic-, RNA-, metabolomics-, and protein-based markers. Finally, the landscape of available meningioma-specific animal models is overviewed. Use of these animal models can enable planning of adjuvant treatment, potentially assisting in pre-operative and post-operative decision making. Discovery of novel biomarkers will allow, in combination with WHO grading, more precise meningioma grading, including meningioma identification, subtype determination, and prediction of metastasis, recurrence, and response to therapy. Moreover, these biomarkers may be exploited in the development of personalized targeted therapies that can distinguish between the 15 diverse meningioma subtypes.

## 1. Introduction

Meningiomas are the most prevalent primary intracranial tumors. Meningiomas have an incidence of 7.86 cases per 100,000 persons per year, accounting for around 36% of all central nervous system (CNS) tumors and 53% of nonmalignant CNS tumors [1,2]. Risk factors of meningiomas include radiation therapy, diabetes, genetic susceptibility, arterial hypertension, estrogen use in women, and potentially smoking [3,4]. Nonmalignant meningiomas are more common in women than in men. Meningiomas are also more prevalent in older people and are largely prevalent in the US black population [5]. Arachnoid cap cells, which are found in the thin spider-web-like meningeal membrane that surrounds the brain and spinal cord, are the origin of meningiomas. Most meningiomas are benign and are frequently discovered incidentally [1]. Nearly 80–90% of meningiomas arise intracranially, while the remaining 10–20% arise in the spinal cord [2]. Former and current editions of the World Health Organization (WHO) categorization of tumors of the CNS describe 15 unique meningioma subtypes with heterogeneous physical characteristics encompassing variations in both histological and cytological features. WHO classification of CNS malignancies divides the fifteen meningioma subtypes into three groups: nine types are classified as WHO Grade 1 (benign, low-grade, 80% of all meningiomas), three as Grade 2 (intermediate, high-grade, atypical, 5–15% of all meningiomas, higher chance of recurrence following gross total resection), and three as Grade 3 (malignant, high-grade, anaplastic, 1–3% of all meningiomas, very poor clinical outcomes, and higher possibility of recurrence and metastasis) [6,7,8]. Indeed, there is a huge divergence in individual clinical behaviors of atypical and malignant meningiomas (Grade 2n. Grade 3). The current WHO grading system, which depends mainly on histopathological features, fails to predict outcomes such as recurrence and patient survival in some patients. Therefore, the discovery of reliable meningioma biomarkers is an urgent priority for the prediction of treatment options and a better prognosis of this disease [9].

**Figure 1:**
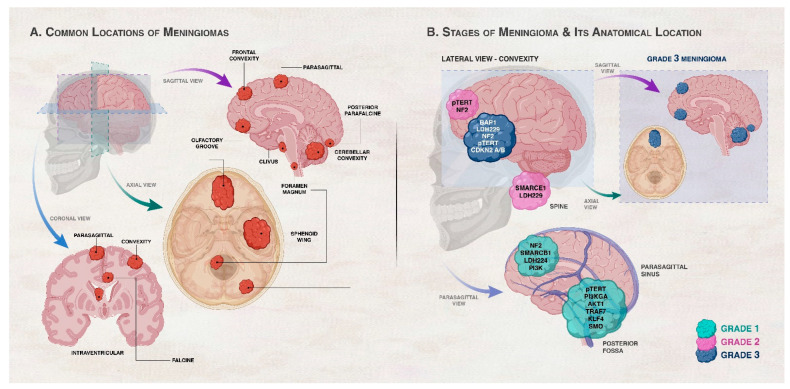
Association between genetic/cytogenetic alteration, grade of meningiomas, and anatomical location of the meningioma. (**A**) shows the common locations of meningiomas in the central nervous system (CNS). Meningiomas arise in the meningeal layers of the brain or spinal cord. They are commonly seen in the parasagittal area, brain convexity, posterior fossa, skull base, and spine. (**B**) illustrates the common locations and gene mutations in meningiomas according to grade. Convexity meningiomas usually harbor *NF2* and SMARCB1 mutations. Brain convexity harbors more Grades 2 and 3 meningiomas than skull base. Skull base meningiomas harbor mutations in AKT1, KLF4, TRAF7, SMO, PIK3CA, and POLR2A genes. Spinal cord meningiomas often harbor SMARCE1 mutations. Locations of Grade 3 meningiomas are highlighted in the right inset of panel (**B**). Grade 1 (benign) meningiomas commonly occur in the parasagittal and posterior fossa with alterations in chromosome 22 and variation in the second allele of neurofibromatosis 2 (*NF2*). Genetic alterations in *AKT1*, *PIK3CA*, *SMO*, *TRAF7*, *KLF4*, and *SMARCB1* also take place in Grade 1 meningiomas in the presence or absence of *NF2* mutations depending on the gene. Grade 2 (atypical) meningiomas tend to exist in the brain convexity and spine and can have a loss of a copy of chromosomes 1, 10, or 14 in addition to genetic alterations in *NF2* and *SMARCEl*. Grade 3 (malignant or anaplastic) meningiomas are characterized by the absence of chromosome 9p and genetic alterations of *NF2*, *BAP1*, *LDH229*, CDKN2 A/B, and *pTERT*. BAP1 mutations are frequent rhabdoid meningioma subtype, rhabdoid meningiomas with BAP1 mutations are more aggressive compared to rhabdoid meningiomas devoid of these mutations [30].

Meningiomas were one of the first malignancies in which cytogenetic abnormalities were discovered. Recent genomic analyses of meningiomas revealed significant molecular variability. In fact, 60–80% of meningiomas have a loss of one copy of 22q, which harbors the neurofibromatosis type 2 (*NF2*) gene, and this loss is usually coupled with alterations of the remaining *NF2* allele [10,11,12]. In fact, up to 60% of sporadic meningiomas have biallelic inactivation of *NF2* due to chromosome 22 monosomy combined with *NF2* point mutations [13,14]. Studies conducted afterwards revealed that the probability of recurrence and malignancy are both correlated with an accumulation of other chromosomal abnormalities, most typically losses of 1p, 10, and 14q [15,16]. In addition to *NF2* mutations, somatic mutations of tumor necrosis factor receptor-associated factor 7 (*TRAF7*), DNA-directed RNA polymerase 2 subunit RPB1 (*POLR2A*), Protein Kinase A Type 1a Regulatory Subunit (*PRKAR1A*), Phosphatidylinositol-4,5-Bisphosphate 3-Kinase Catalytic Subunit Alpha (*PIK3CA*), Kruppel-Like Factor 4 (*KLF4*), AKT Serine/Threonine Kinase 1/Protein Kinase B (*AKT1*), Smoothened Frizzled Class Receptor (*SMO*), Suppressor Of Fused Homolog (*SUFU*), and genes of the transforming growth factor beta pathway (TGFβ) among others have been detected in meningiomas. Some of these mutations may co-occur with *NF2* mutations while others occur independently of *NF2* mutations. Interestingly, some of these mutations are implicated in certain types of meningiomas like those that appear in distinct locations or are of distinct histological subtypes or severity [17,18,19,20,21,22]. Figure 1 demonstrates the relation between genetic alterations, grades of meningiomas, and anatomical location of the tumor in the CNS. However, these somatic driver mutations cannot inform treatment stratification for intracranial tumors [23], and there is an urgent need to understand how these genomic changes are linked to disease outcomes such as tumor recurrence following resection, response to radiotherapy, and overall survival [9].

While genomic markers of meningiomas, like *NF2* mutations, have been explored, the search for other classes of biomarkers is in progress. For example, different WHO grades of meningiomas show differential protein profiles, paving the way for the discovery of protein-based biomarkers [24]. Along the same lines, epigenetic and mRNA biomarkers are currently under investigation in meningiomas. There is evidence that defects in epigenetic regulation are essential for tumorigenesis and that genomic mutations can only partially explain the early stages of tumorigenesis. Indeed, epigenetic alterations of trimethylation of lysine 27 on histone 3 (H3K27me3) repress gene expression and have been implicated in the pathogenesis of intracranial tumors, and loss of H3K27me3 alterations has been associated with meningioma recurrence in retrospective clinical studies [25,26]. In addition, hypermethylation of TIMP3, Cyclin-Dependent Kinase Inhibitor 2A (CDKN2A), and TP73 has been correlated with meningioma grade [27,28]. Ultimately, a panel of meningioma biomarkers combining epigenetics, transcriptomics, proteomics, and genomics biomarkers will be needed to predict behaviors of aggressive meningiomas with a high risk of progression or recurrence [29]. 

In this review, we aim to evaluate genetic and other molecular alterations involved in meningiomas and how to exploit them for new biomarker discovery for diagnosis and prognosis including meningioma identification, grading and subtype determination, and risk of metastasis and recurrence.

## 2. Grading of Meningiomas

The majority of meningiomas (more than 80%) are WHO Grade 1, with Grade 1 age-adjusted incidence rates of 3.68/100,000 and 8.56/100,000 in the male and female populations, respectively [6]. WHO Grade 2 meningiomas have an age-adjusted incidence rate of 0.26 per 100,000 males and 0.30 per 100,000 females. WHO Grade 3 meningiomas are a rare disease with age-adjusted incidence rates of 0.08 per 100,000 males and 0.09 per 100,000 females [31]. Diagnosis of meningiomas is made through imaging, and a biopsy is not necessary if imaging strongly suggests a meningioma [32]. Asymptomatic meningiomas grow linearly at a rate of 2–4 mm per year; however, there can be instances where there is no growth in volume [33]. This aspect highlights the significance of surveillance in untreated patients with asymptomatic meningiomas. Grades 2 and 3 meningiomas are usually symptomatic or have a high tendency for growth and undergo gross total resection [34]. Occasionally, not all the tumor is accessible for resection leading to recurrence. It has been observed that the extent of resection affects recurrence rates [35]. The estimated 10-year overall survival for benign meningiomas is 81.4%, compared to 57.1% for malignant ones. Grade 2 tumors’ 10-year overall survival rate is around 53%, while Grade 3 tumors sadly have this rate as 0% [2]. Meningiomas with distant metastasis are rare and have only been documented in few case reports or brief case series [36,37,38,39]. The lungs, bones, spinal cord, and liver are the most common secondary metastasis sites of meningiomas [36]. Only 6% of metastases are discovered at the time of diagnosis, while 93% of metastatic meningiomas are discovered after the main tumor has been diagnosed and removed [36].

Recent developments in genomics have led to further stratification of meningioma subtypes based on alterations in somatic gene copy numbers and genome-wide profiling of DNA methylation [20,40,41]. Patel et al. combined whole-genome sequencing and transcriptome analysis and suggested the classification of meningiomas into three major types: type A includes missense mutations in *TRAF7*, *KLF4*, and *AKT1* and has minimal chromosomal alterations [42], similar to previous findings in benign meningiomas [40]; type B includes *NF2*-deficient non-aggressive meningiomas; and type C includes more aggressive meningiomas, which have a significant chromosomal instability and chromosomal gains and losses, most commonly losses of both chr22q and chr1p [42]. Using these molecular principles, Tsitsikov et al. compared transcriptional profiles of four of the most common benign types of meningiomas: (1) *NF2* loss versus meningiomas with *TRAF7* missense mutations, (2) *NF2* tumors with or without additional loss of chr1p, and (3) *TRAF7* meningiomas with additional missense mutations in *AKT1* or *KLF4*. Their analysis showed distinct transcriptional programs specific for each meningioma genotype [40]. Other studies have integrated multiple parameters, including DNA methylation, RNA-seq, and cytogenetic profiling to enhance the grading of meningiomas [43,44]. The significant differences in the molecular profiles between the different meningioma grades led to the recognition of certain high-risk molecular signatures in the WHO 2021 classification of CNS tumors [8]. In this WHO classification, loss of H3K27me3 is indicative of aggressive meningioma behavior and recurrence, and homozygous deletions of *CDKN2A/B* and mutations of *TERT* promoter (*pTERT*) are criteria for Grade 3 meningiomas since they are linked to an increased risk of recurrence [8,45,46]. However, these added molecular markers can specify only a subtype of meningiomas that are at high risk of recurrence. This further underscores the need to include more molecular markers for meningioma identification and that meningioma grading should not depend on histopathology only [46].

## 3. Genomic Alterations and Epigenetic Modifications in Meningiomas

**Figure 2:**
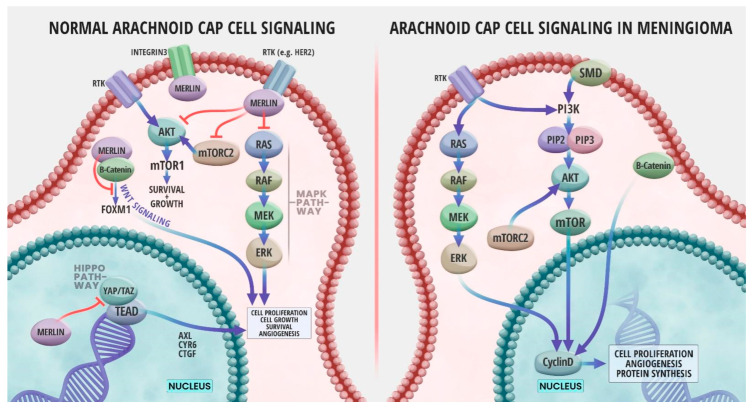
*NF2*/Merlin signaling in a normal meningeal cell vs. Merlin-deficient meningioma cell. Merlin is an effective inhibitor of major signaling pathways that lead to cell proliferation, protein synthesis, and angiogenesis. In a normal meningeal arachnoid cap cell, the *NF2* gene encodes for Merlin. Merlin is a cytoskeletal protein that interacts and complexes with integrin 3, receptor tyrosine kinases (RTKs), and β-catenin to inhibit mTOR signaling pathway, MAPK pathway, and WNT pathway, among others. Merlin inhibits downstream effectors of these pathways including RAS, PI3K, AKT, mTOR, and β-catenin. Additionally, Merlin interferes with the translocation of β-catenin into the nucleus, inhibiting canonical WNT signaling. Merlin also inhibits transcription factors YAP/TAZ and TEA by interacting with components of the Hippo pathway. Loss of Merlin function to *NF2* mutations, such as in meningiomas, activates these pathways (indicated by arrows) and leads to cell proliferation, protein synthesis, and angiogenesis, contributing to meningioma incidence and progression.

Advances in technology over the last few decades have led to an ongoing rapid growth in the understanding of the oncogenesis and genomic profiles of meningiomas. One outcome of such advances was the association between meningioma formation and *NF2* gene inactivation. Later, genomics studies identified numerous meningioma genetic alterations, many of which were not in the *NF2* gene [47]. *NF2* is named after neurofibromatosis type 2, which is a genetic condition in which benign tumors grow along the nerves responsible for hearing and balance; mutations in the *NF2* gene were found to cause the disease. The *NF2* gene is located on chromosome 22q12.2 and codes for a 69 kDa protein, Merlin [48]. Merlin protein can be found in a variety of adult and embryonic human tissues, specifically in Schwann, meningeal, lens, and nerve cells. Merlin is a cytoskeletal protein that functions in crosslinking membrane proteins with the cytoskeleton [48]. Loss of the Merlin protein interrupts normal cell growth by creating gaps in adherens junctions [49]. Merlin is known to act as a tumor suppressor by inhibiting cell growth through contact inhibition and activation of multiple signaling pathways [50], and genetic inactivation of *NF2* prevents the production of Merlin, leading to meningioma formation [28]. Figure 2 illustrates *NF2*/Merlin signaling pathways in a normal arachnoid cap cell in comparison to an *NF2*-deficient meningioma cell.

*NF2* is the most recurrently mutated gene in sporadic and radiation-induced meningiomas [51]. Merlin inactivation, due to mutations in *NF2*, is involved in about half of sporadic meningiomas [50]. In fact, 60% of meningiomas have been characterized by an *NF2* gene deficiency caused by promoter methylation, epigenetic inactivation, monosomy of chromosome 22, or a somatic mutation [52]. Low expression of Merlin was associated with tumor recurrence and worse overall survival and progression-free survival (PFS) in large patient studies [53,54]. These studies suggested that the mutation status of *NF2* can act as a biomarker of the survival, prognosis, and risk of tumor recurrence in meningioma patients.

The presence of *NF2* mutations is the basis for the classification of meningiomas into a subtype that has *NF2* gene alterations and a subtype associated with non-*NF2* somatic mutations [47]. Meningiomas with mutations in non-*NF2* genes are less common, more heterogeneous, and often result in different tumor phenotypes [22,55]. Indeed, missense mutations in TRAF7, KLF4, and AKT1 exist in 30%, 14%, and 12% of non-*NF2* meningiomas, respectively [54,56]. Studies have identified driver *TRAF7* somatic mutations in meningioma tumorigenesis [22,55]. These are the most common non-*NF2* mutations and are detected in over 30% of non-*NF2*, Grade 1 tumors, whereas Grade 3 tumors were less likely to result from these mutations. *TRAF7* mutations are exclusive from *NF2* mutations, suggesting that the two genes act along the same pathway. Additionally, *TRAF7* mutations instigate meningioma growth by acting in combination with one of various co-mutations such as *KLF4* and *AKT1*. *KLF4* and *AKT1* mutations co-exist with *TRAF7* mutations but not with each other [22,55,56]. KLF4 is a transcription factor that regulates differentiation in a variety of cell types, and its expression is essential to reprogram adult cells into adult pluripotent stem cells while AKT1 is involved in proliferation signaling and is a well-characterized oncogene [57].

In addition, there are more rare germline mutations in meningiomas including mutations of Switch/Sucrose non-Fermentable Family (SWI/SNF)-Related, Matrix-Associated, Actin-Dependent Regulator of Chromatin, Subfamily B, Member 1 (*SMARCB1*), *SMARCE1*, *BAP1*, and *SUFU* genes. *SMARCB1* and *SMARCE1* mutations are frequently reported in familial syndromes with multiple meningiomas [47]. Mammalian SWI/SNF complex is a multi-subunit chromatin remodeling complex that uses the energy of ATP hydrolysis to remodel nucleosomes and regulate DNA accessibility in fundamental cellular processes, such as transcription and DNA replication and repair. Mutations of components of SWI/SNF complex are frequently observed in numerous human cancers; however, the underlying mechanisms by which SWI/SNF components contribute to tumorigenesis or drug sensitivity warrant further investigation. It also remains unknown whether and how SWI/SNF mutations or defects could be exploited for therapeutic purposes [58].

Epigenetic modifications are major regulators of gene expression, and there is evidence that abnormalities in epigenetic regulation are a critical part of the process of tumorigenesis. Modification of DNA methylation profiles is one of the best-characterized epigenetic alterations implicated in carcinogenesis. Cancer cells usually undergo a global hypomethylation of their genomes, with only selected regions around promoters of specific genes undergoing DNA hypermethylation. The altered DNA methylation profiles cause alterations in gene expression [47,59]. Conserved CpG islands next to gene regulatory elements in cancer cells exhibit DNA hypermethylation and gene silencing, which correlate with tumor aggressiveness and recurrence. These abnormal changes in DNA methylation are usually unique and can be exploited to characterize a cancer type [47,60,61]. Indeed, methylation profiles of specific genes were shown to correlate with a shorter time to meningioma recurrence [62]. These results can be used to predict prognosis and guide the selection of therapeutic options. As mentioned, loss of H3K27me3 modifications has been associated with meningioma recurrence in retrospective clinical studies [25,26], and hypermethylation of *TIMP3*, *CDKN2A*, and *TP73* has been correlated with meningioma grade [27,28]. Hypo- and hypermethylation of numerous other genes have been correlated with the severity, recurrence, and metastasis of meningiomas, as has been reviewed in [60,63] and Table 1. Changes in DNA methylation patterns can be combined with the existing molecular biomarkers to further classify meningiomas into subtypes of different severity and potential for recurrence or metastasis [60]. Choudhury et al. developed a tool, Meningioma Methylation Classifier (https://william-c-chen.shinyapps.io/MeninMethylClassApp/, accessed on 5 November 2023), which classifies meningiomas according to their DNA methylation status [64]. As proposed by Singh et al., genome-wide DNA methylation profiling represents a paradigm shift in meningioma classification, prognostic prediction, and treatment strategy [63].

Recently, metabolomic biomarkers are emerging as promising candidate biomarkers to stratify meningiomas. Several studies have identified metabolomic signatures that may differentiate between meningioma grade, aggressiveness, and recurrence risk (Table 1).

## 4. *NF2*/Merlin Signaling Pathways in Meningiomas

Merlin is known to interrupt cellular growth by signaling through several cellular signaling pathways (Figure 2), such as inhibition of the RAS/RAF/MEK/ERK mitogen-activated protein kinase (MAPK) signaling pathway, which is relevant to organism growth and development and survival of cells [92]. In normal meningeal cells, Merlin forms a complex with the receptor tyrosine kinase human epidermal growth factor (ERB B2, HER2) and integrin β1 at the cell membrane. This complex inhibits protein kinase B (AKT) and extracellular signal-regulated kinase (ERK) MAPK by preventing the accumulation of ERB B2 and ERB B3 (HER3), two members of the epidermal growth factor receptor (EGFR) family (Figure 2). Merlin can also act upstream of the RAS/RAF/MEK/ERK pathway by inhibiting activation of RAS and RAC following growth factor stimulation (Figure 2) [93]. Merlin deficiency due to *NF2* mutations often results in the overactivation of the RAS/ERK pathway, therefore leading to tumor development (Figure 2) [50]. It is common for RAS expression to be elevated in patients with meningiomas. Furthermore, the extent of the RAS increase could serve as an index for determining the degree of malignancy and grade of the meningioma [92]. A study analyzing the expression of various signaling proteins in 70 primary meningiomas indicated strong immuno-expression of RAS and RAF in almost all Grade 1 meningiomas. However, the expression of RAS and RAF was decreased in Grades 2 and 3 meningiomas, suggesting that these tumors might have other dysregulated pathways than that of RAS/ERK. Additionally, the same study found that RAF was associated with meningioma recurrence, thus highlighting the importance of the RAS/RAF/MEK/ERK pathway activation for meningioma growth [94]. Furthermore, animal models have shown that inhibition of Ras activity suppresses proliferation and induces apoptosis of meningioma cells [92], suggesting that Ras might be an ideal target in meningioma treatment. However, further research is needed on the dysregulation of the RAS/ERK pathway in meningiomas.

Merlin has also been reported to signal through the Hippo tumor suppression pathway (Figure 2), the main pathway of cellular growth and regulation of organ/tissue size. The mechanism by which Merlin regulates upstream signals of this pathway are not fully understood yet. However, it is known that loss of Merlin lipid binding ability severely compromises Hippo pathway. *NF2* mutants that result in a Merlin protein deficient in phosphoinositide binding prevent osmotic stress-induced activation of the Hippo pathway [95]. Experiments in Drosophila and mice as well as in vitro using human cells have shown that *NF2* acts through this pathway to keep tissue growth in check. Deletion of *NF2* in human cells was sufficient to completely abolish the Hippo pathway response to glucose starvation, actin disruption, or serum deprivation [96]. Inactivating mutations of the *NF2* gene inactivates the Hippo pathway, allowing the transcription factors Yes-associated protein (YAP)/Transcriptional coactivator with PDZ-binding motif (TAZ) to move into the nucleus and form a complex with TEAD (TEA domain) transcription factor, thus promoting cell proliferation and preventing apoptosis by activating the transcription of genes such as AXL Receptor Tyrosine Kinase (*AXL*), Cysteine-Rich Angiogenic Inducer 61 (*CYR61*), and Connective Tissue Growth Factor (*CTGF*). A study involving the analysis of 57 meningiomas demonstrated a significant elevation of expression of these Hippo pathway-associated genes, in tumors involving *NF2* mutations, but without any correlation with the grade of the meningioma [95]. Indeed, high levels of YAP1 were found to have nuclear localization in meningiomas, and targeting YAP1 activity was shown to be a potential treatment option in meningiomas [97]. Furthermore, YAP undergoes frequent modifications, often through its fusion with other proteins, such as *MLM-2*, *MAML2*, *PYGO1*, and *LMO1*, in meningiomas and other types of tumors linked to neurofibromatosis type 2 [98]. These provide evidence that Hippo pathway dysregulation is a common driver of oncogenesis in meningiomas and other rare cancer types of the CNS [98]. TEAD palmitoylation inhibitors prevented the growth of *NF2*-null schwannoma and *NF2*-null meningioma cells in vitro and in a mouse model [99]. Similarly, constitutive activation of YAP1 or the presence of YAP1-MAML2, a fusion protein that was identified in several meningioma patients, can drive the formation of tumors that resemble *NF2* mutant meningiomas [98]. Despite current evidence suggesting that Hippo pathway *YAP* fusion events may act as alternative drivers of meningioma incidence than *NF2*, further research is needed to understand the oncogenic functions of the Hippo pathway in meningiomas in order to exploit these functions in diagnosis and the discovery of specific therapeutic targets for treatment of meningiomas and other tumors.

Merlin interacts with the phosphoinositide 3-kinase/AKT/mammalian target of rapamycin (PI3K/AKT/mTOR) signaling axis (Figure 2), which contributes to the regulation of cell growth and proliferation [100]. Activation of PI3K by a growth factor, for example, will cause phosphorylation and activation of AKT, which can activate mTOR complex (mTORC), allowing for the translation of mTOR target proteins [100]. Merlin inhibits the activation of PI3K by binding phosphatidylinositol 3-kinase enhancer-L [101]. The PI3K/AKT/mTOR axis is overactive in meningiomas [102]. Activating mutations of AKT were identified in a subtype of meningiomas [102], and high-grade meningiomas have higher expression levels of AKT, which support a role for PI3K/AKT in meningiomas [94,102]. High levels of active phosphorylated mTOR were associated with shorter PFS and increased recurrence in atypical meningiomas [103]. Merlin negatively regulates mTORC, whereas Merlin-deficient meningioma cell lines and tumors show constitutive activation of mTORC1 [104]. Merlin-mediated inhibition of mTROC is PI3K/AKT- and ERK MAPK-independent implying the existence of a non-canonical mechanism of mTORC1 inactivation by Merlin [102]. This mechanism remains unexplored and requires further research. Inhibitors of mTORC1 were tested using in vitro, in vivo in animal meningioma models, and in patients and were shown to significantly reduce the proliferation of meningioma cell lines and animal models [102]. Moreover, the combined inhibition of mTORC and angiogenesis increased overall progression-free survival to 22 months in 17 patients with progressive or refractory symptomatic meningiomas [102,105]. Similarly, mTORC inhibition was safe and extended the PFS of 28 patients with recurrent or progressive Grades 2–3 meningiomas in a phase II trial [106].

Merlin also acts as a negative regulator of the forkhead box M1 (FOXM1)/WNT signaling pathway (Figure 2). The WNT signaling pathway is essential during embryogenesis and CNS development and is known to be associated with cancer cell growth and rapid tumor development [52,107]. Components of WNT signaling regulate multiple aspects of brain development in vertebrate embryos. WNT signaling leads to the accumulation of the transcription factor β-catenin in the cytoplasm and its subsequent translocation to the nucleus. Relatedly, mutations in the *β-catenin* gene have been reported in a variety of human tumors [107]. A study by Lau et al. illustrated a relationship between Merlin and WNT signaling in human glioma cells where re-expression of Merlin reduced WNT signaling. The levels of WNT receptor Frizzled-1 (FZD1) were reduced, and the expression of molecules that inhibit WNT signaling, Dickkopf-1 (DKK1) and Dickkopf-2 (DKK2) were increased [108]. Additionally, hypermethylation and inhibition of polycomb repressive complex (PRC) that causes *NF2* mutations have been shown to potentiate WNT signaling. Mutated *NF2* serves as a functional switch for *FOXM1* transcription. Overexpression of FOXM1 due to the lack of regulation by Merlin promotes meningioma cell proliferation and viability. FOXM1 interacts with β-catenin to increase WNT signaling [52].

Overall, signaling pathways that are affected by Merlin loss of function continue to emerge as possible targets for therapy [93]. However, much remains unknown in regards to the exact mechanisms by which these pathways influence meningioma grading, pathology, and prognosis.

## 5. Biomarkers of Meningiomas

### 5.1. Current Diagnosis and Prognosis

Current approaches for the diagnosis of meningiomas rely on patient medical history, physical examination, and use of radiological techniques like computed tomography (CT) scans and magnetic resonance imaging (MRI). MRI remains the gold standard for radiologic diagnosis and is also used for long-term follow-up as there is no exposure to radiation [34,109]. However, in cases where MRI is counter-indicated, such as in patients with pacemakers, contrast-enhanced CT scans are used [110]. The challenge in using radiology to diagnose meningiomas is the similarity of meningiomas to other intracranial lesions in MRI and CT scans, complicating diagnosis. Figure 1 depicts the grades of meningiomas and their anatomical locations in the CNS where other CNS tumors may also arise, further complicating diagnosis. For example, in the diagnostic process, whenever a suspected meningioma is encountered, the possibility of it being a hemangiopericytoma is also considered. Meningiomas originate from meningothelial cells (arachnoid cap cells), while hemangiopericytomas arise from pericytes, which are cells found in close proximity in the blood vessels. Furthermore, meningiomas that are present in the cerebral hemispheres can be challenging to distinguish from dural (pachymeningeal) metastases, particularly metastases of prostate, lung, kidney, or breast cancers, primary glial tumors that extend into the subarachnoid space, and hematopoietic neoplasms like extra-axial non-Hodgkin lymphoma [111,112,113,114]. Meningiomas at the base of the skull, particularly at the cerebellopontine angle, must be distinguished from vestibular and trigeminal schwannomas and neoplastic meningitis. In order for imaging modalities to detect meningiomas, the tumor must grow to a certain size. This becomes another major limiting factor of diagnosis since meningiomas are slow-growing tumors, so the patient remains undiagnosed for early-stage tumors for a long period. For example, fibrous meningiomas and meningothelial meningiomas take an average of 26.3 years and 17.8 years, respectively, until a tumor mass is discovered after the initial cellular change [115]. In meningioma diagnosis, the challenge is not only to confirm the diagnosis of meningiomas but also to identify its subtype and grading. MRI can help in the diagnosis of meningiomas, but it may not be able to distinguish between different meningioma subtypes. Studies have also shown that patient movement during the MRI examination can introduce motion artifacts, compromising image quality and diagnostic accuracy [116,117]. All these challenges involving imaging can be avoided by the use of histopathological assessment, which is becoming the new criterion for the diagnosis of meningiomas [32]. Histological techniques provide static snapshots of tissue morphology, lacking real-time or dynamic information about cellular processes or molecular interactions. However, this involves obtaining a tissue biopsy, which not only is an invasive procedure but also may not be a widely available option. The quality of the biopsy sample, which might occasionally be constrained by tumor location, size, or level of vascularity, can also impact the accuracy of diagnosis [116,117]. Differentiation between different CNS tumor types and meningiomas and meningioma subtype determination and grading require the discovery of new meningioma-specific biomarkers. Collectively, the limitations of MRI and histological techniques highlight the need for new biomarker discoveries to enhance diagnostic accuracy, improve early disease detection, and enable non-invasive monitoring of disease progression.

### 5.2. The Need for a Profile of Biomarkers of Different Types

The need for new meningioma biomarker discovery is underscored by the complex WHO histological diagnostic criteria and the varied morphological characteristics of meningioma subtypes. The complexity is most prominent in WHO Grade 2 tumors, where inter-observer discrepancy can reach 12.2%, as opposed to 7% in Grade 1 and 6.4% in Grade 3 tumors [118,119]. Grade 2 tumors can behave biologically similarly to Grades 1 or 3 tumors with unexpected clinical outcomes due to their very diverse histological characteristics [26,120]. Furthermore, Grade 1 meningiomas that are clinically aggressive can also have clinical outcomes resembling those of Grade 2 tumors [121]. These uncertainties make it clear that imaging and classical histological techniques alone cannot be used to predict the prognosis and clinical course of meningiomas and further highlight the need for the discovery of novel meningioma biomarkers. These novel biomarkers can assist in the diagnosis, management, and prognosis of meningiomas given the growing emphasis on an integrated molecular approach to diagnosing CNS tumors [30,122]. Currently, there is a lack of non-invasive meningioma diagnostic or prognostic biomarkers. These biomarkers may have an impact on the early detection of meningiomas, patient management, and clinical outcomes [123,124].

Proteomics, metabolomics, epigenomics, metabolomics, RNA sequencing (RNA-seq), and single-cell RNA-seq (scRNA-seq) are emerging approaches that have aided in the discovery of new biomarkers for several diseases and ailments. These biomarkers include specific molecules, genetic variations, or imaging characteristics that are associated with the presence, severity, or progression of diseases [46,125,126]. They may offer an opportunity to develop more accurate diagnostic tests, predict treatment responses, identify therapeutic targets, and monitor disease progression in a non-invasive manner. Marastoni and Barresi have most recently reviewed the potential of these emerging technologies in comparison to histopathological markers and WHO grading. They compared meningioma grading based on meningioma methylation status in several studies and concluded that DNA methylation profiles are more accurate predictors of meningioma prognosis than the WHO grading system [46]. In this regard, Kishida et al. first reported that recurrent meningiomas have a greater number of methylated genes in comparison with nonrecurrent meningiomas, indicating the prognostic potential of DNA methylation profiles in meningioma grading [127]. Later, Olar et al. reported that among a training cohort of 89 tumors and a validation set of 51 tumors, prognostically unfavorable high-grade meningiomas have more methylated genes, chromosomal CNVs, and shorter recurrence-free survival than prognostically favorable low-grade meningiomas [128]. Sahm et al. generated genome-wide DNA methylation profiles of 497 meningioma samples and concluded that DNA methylation profiling could distinguish six different clinically relevant methylation classes that also showed differences in mutational, cytogenetic, and gene expression patterns. They also indicated that classification according to these six methylation classes was more accurate than 2016 WHO grading at defining WHO Grade 1 meningiomas at high risk of progression and WHO Grade 2 meningiomas at lower risk of recurrence [129]. Nevertheless, the higher prognostic values of DNA methylation profiles have not been applied in routine diagnosis due to high cost and the requirement of complex technologies [46]. This further emphasizes that newly discovered biomarkers cannot be used independently but need to be integrated into the WHO grading system.

To build on the success of meningioma grading using a combination of DNA methylation patterns and genetic alterations, an integrated molecular–morphological grading approach for meningioma grading was employed [46]. Maas et al. developed an integrated meningioma grading system based on the following determinants: 2016 WHO grade, combined classes of DNA methylation patterns, genetic mutations, and chromosomal copy number changes in chromosomes 1p, 6q, and 14q. A score was given to each of the determinants. The minimal score of all determinants was zero and the maximal score was nine and a score of 0–2 indicated low-risk, a score of 3–5 indicated intermediate-risk, and a score of 6–9 indicated high-risk meningiomas. The integrated grading system was superior at predicting recurrence risk of meningiomas than 2016 WHO grading, combined methylation classes, or chromosomal copy number changes when validated in a set of 471 meningiomas [90]. Relatedly, Driver et al. designed another integrated grading scheme incorporating mitotic count, and loss of chromosomes 1p, 3p, 4, 6, 10, 14q, 18, 19, or CDKN2A was also shown to more accurately identify meningiomas PFS and risk for recurrence, relative to WHO grading [91].

More recent studies have demonstrated that the best approach distinguishing between three biologically distinct categories of meningiomas is to use an integrated molecular grading scheme by combining data from different kinds of biomarkers including somatic DNA point mutations, DNA methylation classes, transcriptomics, RNA-seq, and chromosomal instability (CIN)/cytogenetics [42,43,44,62]. Patel et al. studied 160 meningiomas covering the spectrum of the three WHO categories, which were subtyped using whole-exome sequencing (WES), RNA-seq, and cytogenetics [42]. Three types were delineated: type A rarely recurring malignancies that carry mutations in *TRAF7*, *AKT1*, or *KLF4* but do not exhibit chromosomal deletions; type B meningiomas that lack the chromatin-modifying enzyme PRC2 and are deficient in the *NF2*/Merlin protein; and type C, which is both *NF2*-deficient and marked by CIN, notably the loss of chromosome 1p, and this type has worse recurrence rates [42,44]. Additionally, Nassiri et al. identified integrative molecular groupings using a multi-omics method by incorporating an investigation of somatic DNA point mutations, DNA methylation, mRNA levels, and somatic chromosomal copy number aberrations [43,60]. Interestingly, they discovered four molecular clusters that, in contrast to WHO grading, independently correlated with recurrence-free survival and offered more accurate predictions of time to recurrence than WHO grading [43,60]. In confirmation, Choudhury et al. profiled 565 meningiomas and combined DNA methylation patterns with genetic, transcriptomic, biochemical, proteomic, and single-cell analyses and obtained similar results, showing that meningiomas exhibit three DNA methylation classes with different clinical outcomes, biological drivers, and therapeutic vulnerabilities [62]. In this study, meningiomas were segregated into Merlin-intact meningiomas (34%, best clinical outcomes and response to cytotoxic drugs, owing to the apoptotic function of the intact Merlin protein), immune-enriched meningiomas (38%, have intermediate prognosis, are distinguished by immune cell infiltration, HLA expression, and lymphatic vessels, and have 22q loss and inactivation of *NF2*), and hypermitotic meningiomas (28%, have the worst prognosis, high aneuploidy with frequent chromosomal losses, loss of CDKN2A/B, hypermethylation, and resistance to cytotoxic drugs) [62]. Comparative genome hybridization was also used for the identification of chromosome 1p loss in radiation-induced meningiomas, a less prevalent late danger of cranial irradiation, which has a higher recurrence rate and pathologically malignant characteristics than sporadic meningiomas [130]. A study of 31 meningioma cases, using exome, epigenome, and RNA-seq analyses, revealed the presence of *NF2* rearrangements in radiation-induced meningiomas, and this may be utilized to differentiate this type of meningioma from sporadic ones [131]. One study developed a meningioma progression score (MPscore) to quantify the likelihood of progression in meningiomas and generalize this discriminative ability [132]. Accordingly, the MPscore served as a reliable surrogate for subtype 3 meningioma advancement, conveying that MPscore of subtype 3 was considerably higher than the MPscores of other subtypes [132]; hence, the meningiomas’ recurrence-free survival rate and MPscore were highly correlated. It may be possible to create significant phenotypic meningioma profiles using non-invasive analysis to forecast tumor genetics and behavior. These profiles can then be used to guide non-invasive treatment and management decisions. Wang et al. pioneered the use of scRNA-seq analysis to study immune and non-immune cell types in tissues from non-tumor-associated dura versus primary meningioma tumor tissues of patients, revealing that the human dura has a complex immune microenvironment that is transcriptionally different from that of meningiomas [133]. One pilot study integrated machine-learning methods with bioinformatics techniques to categorize glioblastoma (GBM) subtypes associated with bevacizumab responsiveness based on existing miRNA profiling datasets [134]. This lays out new strategies that may be applied in meningioma biomarker identification to help classify, monitor, and provide therapeutic decisions in meningioma tumors. A newer emerging non-invasive methodology employed a zinc oxide nanowire-based device that can be used to extract a substantially higher diversity and quantity of miRNAs from urine, suggesting that urinary miRNA profiles are suitable for non-invasive CNS tumor mass screening since urinary miRNA expression has been correlated with the incidence of certain tumors [135].

Ongoing research in meningioma biomarker identification aims to integrate all these emerging molecular approaches to define an integrative set of new biomarkers that can non-invasively diagnose meningiomas and stratify the different subtypes of meningiomas. This can serve for a better prognosis of meningiomas and the discovery of new therapeutic targets. Overall, the new integrated molecular approaches [42,43,44,62] have higher accuracy in predicting prognosis and risk of recurrence than 2016 or 2021 WHO grading systems or methylation-based classifications [46]. Based on these new integrated meningioma grading approaches, Marastoni and Barresi conclude their review by defining three meningioma classes, which can complement WHO grading for the prediction of prognosis. Group 1 meningiomas have the best prognosis, are free of *NF2* mutations and chromosomal instability, may include *AKT1*, *TRAF7*, or *KLF4* mutations, and are predicted good responses to cytotoxic therapies. Group 2 meningiomas have intermediate prognosis, *NF2* inactivation, are free of chromosomal instabilities, and are enriched in immune cells. Group 3 meningiomas have the worst prognosis and high chromosomal instability and proliferation indices, show resistance to cytotoxic therapies, and may have *pTERT* mutations and/or *CDKN2A/B* deletion. Although these new classifications were not part of the 2021 WHO meningioma grading, they are expected to guide meningioma grading in the near future. Application of these new grading schemes in clinical practice may face difficulties, but new proteomic studies have indicated that meningiomas may be classified using specific immunostaining targets that can replace the need for sophisticated methods like profiling of DNA methylation or RNA-seq [46].

### 5.3. Exploring Protein Biomarkers as Meningioma Biomarkers

A panel of meningioma biomarkers incorporating proteomics may be able to predict aggressive meningiomas with a high risk of metastasis or recurrence. However, challenges of identifying proteomics-based predictive, prognostic, and monitoring biomarkers go beyond detection of the prevalence of the disease and must in addition consider the type of targeted therapy, response rates to therapy, and time to event analysis, including progression-free survival and mortality [136]. For future research, overcoming these biological and technical difficulties is essential and should be considered throughout the design phase of discovery, during biomarker development, and should be confirmed using distinct validation cohorts [136]. Interestingly, protein-based diagnostic biomarkers may be used as theranostic biomarkers where the protein biomarker is combined with therapeutic agents, such as radioactive compounds [137]. For example, somatostatin receptor subtype 2 (SSTR2) mRNA is overexpressed by all subtypes of meningiomas; therefore, somatostatin peptide analogues (SSTas) have been labeled by different radionuclides for the detection of meningiomas using positron emission tomography (PET) imaging as well as therapy that has been termed targeted peptide receptor radionuclide therapy (PRRT). Using PRRT with SSTa, Saglues et al. were able to prolong the 6-month progression-free survival of progressive refractory WHO Grades 1 and 2 meningiomas, but not aggressive WHO Grade 2 tumors [138]. Another study reported that prostate-specific membrane antigen (PSA) protein expression increases as meningiomas progress in grade or as a result of recurrence and that 98.9% of 91 included meningioma samples express PSA in endothelial cells. The study proposed PSA as a potential theranostic marker of meningiomas [139]. Large-scale randomized trials are needed for the transformation of potential theranostic biomarkers into clinical practice guidelines.

- Serum Protein Biomarkers

There are no blood biomarkers that currently exist for meningiomas, and the discovery of non-invasive protein biomarkers in the serum of patients is a major area of interest in meningioma diagnosis. A serum biomarker can be any substance that changes measurably in the serum as a tumor develops [140], hence it should be able to detect the presence of meningiomas and determine their grades and subtypes. Typically, these biomarkers should be highly expressed on the surface of circulating malignant cells or shed into the blood stream by tumor cells [140]. Using an immunoassay-based detection, it was shown that a panel of seven serum proteins (caspase-3, CD69, prolactin, epidermal growth factor (EGF), chemokine (C-C motif) ligand 24 (CCL24), amphiregulin (AREG), and heparin-binding EGF (HB-EGF)) were strongly expressed in Grade 1 meningioma samples, with caspase-3 emerging as the highest differentially expressed protein [82]; however, vascular endothelial growth factor D (VEGFD), transforming growth factor (TGF-α), E-Selectin, B-cell activating factor (BAFF), interleukin-12 (IL-12), chemokine CCL9, and growth hormone (GH) levels were downregulated [82]. This coincides with the results of a previous study that reported elevated caspase-3 immunoreactivity in Grade 2 and Grade 3 meningioma tissues and proposed caspase-3 as an independent unique predictor of early recurrence [141]. Meningiomas have been linked to the activation of complement cascades by increasing the expression of a few complement (C) components, including C5, C8 beta chain, C6, and C4-B [65]. Particularly, C3, a key protein in tumorigenesis of meningiomas, was found to be downregulated in Grade 2 meningiomas when compared to Grade 1 [142]. Moreover, elevated levels of proteins involved in blood coagulation and hemostasis, such as antithrombin-3, alpha-2-antiplasmin, vitamin K-dependent protein S, fibrinogen alpha chain, plasminogen, alpha-2-macroglobulin, and coagulation factor ×2, were associated with different grades of meningiomas [65].

Hypoxia markers in serum can be potentially used in the diagnosis of meningiomas. Hypoxia is a common feature of many malignant neoplasms. In hypoxia, the transcription factor hypoxia-inducible factor 1 (HIF-1) binds to hypoxia response elements (HREs) and regulates the expression of hypoxia-responsive genes, thereby coordinating many of the responses to hypoxic stress. HIF-1 target genes include the angiogenic factor VEGF, erythropoietin (EPO), glucose transporter-1 (GLUT1), and several glycolytic enzymes, which contain HREs in their promoter or enhancer regions [143]. In a study by El-Benhawy et al., serum levels of hypoxia markers HIF-1α, VEGF, and lactate dehydrogenase (LDH) were considerably decreased after radiotherapy in meningioma patients [144]. Previous studies have demonstrated that acidic pH increases angiogenesis and migration of glioma stem cells by activating glioma stem cell markers [145]. This opens the question of whether elevated LDH levels and acidic pH could also be related to meningioma progression. According to another study, the expression of the endogenous hypoxia marker carbonic anhydrase 9 was highly expressed in more than 50% (29 of 62) of the included meningioma patients, had an expression that was substantially related with higher grade histology, and was prevalent in recurrent tumors [85].

Endocan is another potential serum biomarker of meningiomas. Endocan serum levels were found to vary in relation to meningioma grade; the higher the meningioma grade, the higher the endocan serum levels [146]. These results confirm results of a previous study that tested the levels of endocan in glioma and meningioma brain tumors and concluded that the levels of endocan are increased in tumors of glioma and meningioma patients and the amount of increase correlated with the degree of malignancy [147].

b. Cerebrospinal Fluid Protein Biomarkers

The blood–brain barrier prevents brain tumor-specific molecules from being released into blood circulation, and this limits the number of biomarkers in serum of CNS tumors [148]. As a result, cerebral spinal fluid (CSF) has been investigated for its potential use in the diagnosis of brain tumors [148]. Indeed, oncologists clinically use CSF protein biomarkers because of their utility not only in diagnosis but also in the treatment and evaluation of recurrent malignancies [81]. Brain ventricles are filled with CSF, which also encircles the brain and bone marrow in the subarachnoid space [81], so it is directly in contact with the extracellular environment of the CNS. Hence, CSF cytology is amenable to collection, and lumbar puncture is a non-invasive way of collecting CSF [81]. In one investigation, two-dimensional (2D) gel electrophoresis and mass spectrometry (MS) analysis of CSF samples allowed the identification of upregulated meningioma-specific CSF proteins. The upregulated proteins included apolipoprotein E (APO-E), alpha-1-antitrypsin, and prostaglandin synthases (Table 1) [81,149]. APO-E is present in normal human tissue as well as intracranial neoplasms, and APO-J has anti-amyloidogenic function, acting as a prominent carrier protein of soluble circulating amyloids in bodily fluids. Both APO-E and APO-J are considered as potential CSF biomarkers for the detection of meningiomas [81]. Notably, a recent study measured the level of three APO-E peptides (SELEEQLTPVAEETR, LGPLVEQGR, and AATVGSLAGQPLQER) in meningioma CSF samples, and the results indicated a 2.21-fold increase in APO-E in Grade 2 as compared to Grade 1 meningiomas [142]. On the other hand, ApoA-I, a multifunctional protein involved in regulating immune responses as well as cholesterol transport [150], was downregulated in meningioma Grade 2 tissue compared to meningioma Grade 1 [142]. Additionally, prostaglandin H2 D-isomerase (PTGDS) has been proposed as a potential biomarker of meningiomas. Kim et al. reported that CSF of meningioma patients had reduced PTGDS expression [81], and a recent study validated that PTGDS had considerably higher expression in Grade 1 meningiomas than in Grade 2 [142]. In the CSF of children with medulloblastoma, another CNS tumor, total prostaglandin D2 synthase levels were reduced by six times, most likely as a result of the host reaction to the presence of the tumor [151]. This sheds light on CSF prostaglandin D2 synthase that could be tested as a potential biomarker of meningiomas.

EGF-containing fibulin-like extracellular matrix protein 1 (EFEMP1) levels in CSF of meningioma patients were considerably higher compared to controls (Table 1) [152]. Similarly, CSF levels of carcinoembryonic antigen (CEA), a protein tumor marker that is frequently elevated in a number of human malignancies, can be used for diagnosing primary and metastatic brain tumors including meningeal carcinomas (Table 1) [148]. A previous investigation reported on the concentrations of three tumor markers, CEA, cytokeratin 19 fragments (CYFRA 21-1), and neuron-specific enolase (NSE), in CSF of 35 lung cancer patients with meningeal carcinomatosis of lung cancer and 35 patients with benign brain tumors [153]. The three markers were significantly higher in the serum and CSF of the meningeal carcinomatosis than in the group with benign disease [153].

### 5.4. LncRNA and miRNA in Diagnosis and Prognosis of Meningiomas

MicroRNAs (miRNAs) are short non-coding RNAs that suppress the translation of proteins, typically by binding to the 3′ untranslated regions (3′UTRs) of target mRNAs [154]. Their transcription is deregulated in several malignancies, and many miRNAs have been recognized as disease biomarkers [154]. Circulating miRNAs have been identified in CSF [155]. Zhi et al. compared miRNA expression profiles of 200 miRNAs between 110 meningioma tumors and 35 “normal” adjacent tissue samples [67]. Three novel miRNAs—miR-29c-3p, miR-219-5p, and miR-190a—were proposed as potential prognostic meningioma indicators (Table 1). Advanced clinical stages of meningiomas were associated with downregulation of miR-29c-3p and miR-219-5p and an upregulation of miR-190a. These miRNAs were also strongly linked with elevated meningioma recurrence rates, suggesting the utility of these miRNAs in predicting recurrence [67]. In a different study, downregulation of miR-331-3p combined with partial resection of meningiomas were found to be the most significant predictive biomarkers. Indeed, miR-331-3p predictive power superseded that of miR-15a-5p (*p* = 0.038), miR-146a-5p (*p* = 0.053), and miR-331-3p (*p* = 0.09) in an enlarged patient cohort [156]. Moreover, Zhi et al. examined the expression of 200 microRNAs in meningioma cells and discovered that miR-17-5p, miR-199a, miR-190a, miR-186-5p, miR-155-5p, miR-22-3p, miR-24-3p, miR-26b-5p, mmiR-27a-3p, miR-27b-3p, miR-96-5p, and miR-146a-5p were significantly upregulated in meningioma cells and acted as oncogenic factors, while miR-29c-3p and miR-219-5p were significantly downregulated in meningioma cells [74]. Particularly, miR-21 [157], as well as miR-219-5p [68], enabled the distinction of the primary meningioma histological types with their expression positively correlated with the clinical stages of meningiomas [68,157]. Similarly, the serum levels of miRNA in meningioma patients were examined and miR-106a-5p, miR-219-5p, miR-375, and miR-409-3p significantly increased, whereas the serum levels of miR-197 and miR-224 were markedly decreased [68]. In a study on tissue samples from 55 patients with atypical meningiomas (43 from a radio-sensitive group and 12 from a radio-resistant group), there were 7 significantly upregulated miRNAs (miR-4286, miR-4695-5p, miR-6732-5p, miR-6855-5p, miR-7977, miR-6765-3p, and miR-6787-5p); while 7 miRNAs were significantly downregulated (miR-1275, miR-30c-1-3p, miR-4449, miR-4539, miR-4684-3p, miR-6129, and miR-6891-5p) in patients resistant to radiotherapy [157]. In a different study, miR-181d expression was found to be higher in meningiomas, and this increase in expression was more pronounced in correlation with the advancement of tumor grade [70]. On the other hand, miR-200a exhibited much lower expression levels in recurrent meningiomas than in initially diagnosed ones [158].

Extracellular vesicles (EVs) are nano-sized, lipid bilayer-enclosed structures released by all living cells. EV cargo includes bioactive molecules, like nucleic acids, proteins, lipids, and metabolites. EVs mediate cell–cell communication and have been shown to have physiologically essential functions as well as pathology-related processes such as in cancer and during viral infection [159]. EV cargoes have been proposed as biomarkers of different diseases, including CNS tumors [152]. EVs were also shown to exist in serum as well as CSF [152,160]. The transcription factor GATA-4 was reported to be overexpressed in malignant meningiomas, where it negatively regulates the expression of miR-497-195 cluster and maintains cell viability [157,161]. MiR-497 levels were found to be reduced in serum EVs derived from patients with high-grade compared to benign meningiomas, due to overexpression of GATA-4 in these tumors [161]. Future research is needed to examine the clinical implications of EV miR-497 in the resistance to treatment exhibited by high-grade meningiomas. These studies also suggest the possibility of using transcription factors and their target miRNAs as new tissue-specific biomarkers for higher grade meningiomas. Finally, future research should investigate CSF as well as serum EVs and their cargoes as non-invasive biomarkers of meningiomas. In this regard, Ricklefs et al. recently demonstrated the diagnostic potential of plasma EVs and indicated that DNA carried by EVs reflects the methylation profiles, mutations, and copy number variations in the meningioma cells from which they are derived [162].

Malignant meningiomas have been shown to be significantly regulated by long non-coding RNAs (lncRNAs). LncRNAs are non-coding genes whose transcripts are more than 200 nucleotides [163]. LncRNAs can bind chromatin, attract protein complexes to modify chromatin states, and subsequently control gene expression [164]. In one instance, lncRNAs can control miRNA function by acting as endogenous miRNA sponges to inhibit miRNA function and consequently block the silencing of miRNA target genes [165]. Differential profiling of patients with different meningioma grades and recurrences revealed that mRNA levels of immunoglobulin superfamily containing leucine-rich repeat 2 (ISLR2), anti-mullerian hormone (AMH), and lncRNA-GOLGA6A-1 exhibited the highest prognostic power to predict meningioma recurrence (Table 1) [72]. Interestingly, ISLR2, AMH, and lncRNA-GOLGA6A-1 transcription is controlled by several transcription factors including KLF4, which is linked to activating mutations of meningiomas [72]. Invasive meningioma-associated transcript 1 (IMAT1) is an lncRNA, which was shown to be expressed more strongly in invasive than non-invasive meningiomas [165]. IMAT1 overexpression significantly increased proliferation and invasion of human meningioma cells expressing KLF4. On the other hand, IMAT1 knockout had the opposite effect, suggesting that IMAT1 lncRNA can severely reduce KLF4 anti-tumor effects [165]. Li et al. found that in malignant meningiomas lncRNA-LINC00702 can operate as an oncogene by controlling the miR-4652-3p/ZEB1 axis and activating the WNT/β-catenin signaling pathway [166]. Further research was conducted by Xing et al. [73] who reported that lncRNA-LINC00460 was highly expressed in meningiomas and increased meningioma metastasis and progression via binding to microRNA-539/MMP-9 [73]. Additionally, other findings showed that maternally expressed gene 3 (MEG3), a well-known lncRNA, was significantly downregulated in meningioma tissues and cells, acting as a tumor suppressor and decreasing the expression of A-kinase anchor protein 12 (AKAP12) by targeting miR-29c to suppress cell cycle, migration, invasion, and proliferation in vitro [71]. Other lncRNAs such as lncRNA-NUP210, lncRNA-SPIRE2, lncRNA-SLC7A1, and lncRNA-DMTN were upregulated in meningiomas [74].

## 6. Animal Models for Discovery of Meningioma Biomarkers

The development of several mouse models of meningiomas has immensely benefited the field of meningioma research. Such in vivo models have provided a better understanding of the underlying biological mechanisms of meningioma pathology. Relatedly, these models were employed as tools for the discovery of various biomarkers that are altered in meningiomas. The first mouse model to be developed for meningioma research was the heterotopic xenograft mouse model [167]. In this model, human immortalized cell lines or patient-derived tumor cells (glioblastoma or meningiomas) are injected subcutaneously into mice. Mixing a basement membrane protein mixture (Matrigel) with meningioma cells prior to injection has proven to increase the success rates of tumor development in mice [168]. The resulting tumors exhibit both immunohistochemical and histological features, which are consistent with meningiomas. However, they lack the key components of the meningioma-specific microenvironment including the CSF, bone, arachnoid, and brain. The orthotopic xenograft model overcomes this limitation through injection of the meningioma cells intracranially into immunocompromised mice. McCutcheon et al. established the first meningioma orthotopic xenograft model, using the IOMM-Lee meningioma cell line and first passage primary cell cultures [169]. Previous studies have described the usage of a wide variety of injection sites and volumes as well as different cell types and numbers during xenografting [170]. The utilization of atypical and malignant meningioma cell lines resulted in very high tumor take rates with almost all immunocompromised mice developing tumors post-injection [170]. Immortalized benign meningioma cell lines produced more heterogenous results, with tumor takes that ranged between 55% and 100% [171]. Closely monitoring tumor take and growth is performed in a simpler manner with heterotopic mice models as compared to orthotopic models. Currently, imaging using small-animal MRI [172] and bioluminescence-based methods [173] are the two main techniques being utilized for tumor monitoring in orthotopic models. It is noteworthy that small-animal MRI is expensive and lacks ready availability.

The anatomy, histology, and genetic driver events in an animal model of a tumor should ideally closely mimic the human tumor. Additionally, the ability to manipulate tumor initiation from different temporal and spatial perspectives is key for the successful establishment of a tumor model. Genetically engineered mouse models (GEMMs) facilitate these features by allowing researchers to extensively edit and manipulate genes [174]. Using the Cre-loxP system in GEMMs allows site-specific DNA modifications such as insertions, deletions, and translocations and has been extensively used in meningioma research following advances in the molecular analysis of human meningiomas [170]. Second-generation GEMMs used for meningioma research introduced modifications to promoter of the prostaglandin-D2-synthase (PGDS) gene to establish meningiomas in mice [175]. In the CNS, PGDS is responsible for prostaglandin D2 biosynthesis and is identified as a marker of meningeal cells in rats, mice, and humans [176,177,178]. Another system used to establish GEMMs is the RCAS-TVA gene delivery system, which is popular for modeling human cancer [179]. Overexpression of the platelet-derived growth factor (PDGF) in arachnoïdal cells using the RCAS-TVA system leads to meningioma development independently of *NF2* mutations [94]. PDGF overexpression combined with the presence of *NF2* mutations and the additional loss of *Cdkn2ab* was shown to induce malignant progression in this model [175]. New GEMMs are needed to improve our understanding of the biological mechanisms involved in meningioma tumorigenesis. This will facilitate pre-clinical drug evaluation as well as the discovery of new specific meningeal markers. The advantages and limitations of the mentioned meningioma pre-clinical models, which can be utilized for biomarkers research, are presented in Table 2.

GEMMs were utilized by Kalamarides et al. to demonstrate that the excision of *NF2* exon 2 in arachnoïdal cells is rate-limiting for meningioma development in mice where 30% of mice developed meningiomas [180]. Meningiomas appeared in mice at four months of age and were histologically similar to human ones. It was also reported that *NF2* and *p53* mutations do not synergize in meningeal tumorigenesis, since disease frequency and progression were not affected with additional *p53* hemizygosity. In a follow-up study, the same authors reported that meningothelial proliferation and meningioma frequency increased, without variations in the tumor grade, in mice nullizygous for the tumor suppressor *p16* (*Ink4a*), revealing a synergy between *NF2* and *p16* inactivation in meningioma development [181]. Another genetic study revealed that meningioma progression in mice was facilitated by a cooperation between *NF2* and *cdkn2ab* [182]. Deleting *cdKn2ab* was associated with shorter latency and an elevated frequency of meningiomas in mice [182].

Interestingly, Mandara et al. investigated steroid receptors in canine and feline meningiomas and revealed that among nine meningiomas from dogs and five from cats that were examined utilizing immunohistochemistry, meningiomas with a high proliferation index exhibited the lowest levels of progesterone receptor (PR) expression [183]. Alterations in estrogen receptor expression were not significant in the investigated samples [183]. In a xenograft mouse model, it was found that PR expression was dependent on the cell cline utilized for injection [184]. In both heterotopic and orthotopic approaches, transplantation of low-passage patient-derived tumor cells formed meningiomas positive for PR and vimentin. However, subcutaneous injection of high-passage cells yielded PR-negative and vimentin-positive tumors, consistent with high-grade meningiomas [184]. An in vivo study utilizing a heterotopic xenograft mouse model demonstrated that FoxM1 is a key transcription factor and oncogenic driver in meningioma progression [185]. The authors injected OMM-Lee cells in control nude mice and in nude mice pre-treated with siomycin A, a FoxM1 inhibitor. Inhibition of FoxM1 resulted in the formation of significantly smaller tumors. Moreover, the knock down of *FOXM1* in meningiomas decreased the number of β-catenin-expressing and Ki67-positive proliferating tumor cells [185]. However, overexpressing *FOXM1* in transplanted benign meningioma cell lines failed to produce tumors in mice, suggesting that FOXM1 alone was insufficient to drive meningioma growth in vivo [185]. The heterotopic xenograft mouse model was also used to explore the role of miR-200a in meningioma tumor growth [186]. Subcutaneous injection of meningioma cell line SF4433-Fluc overexpressing miR-200a into athymic mice resulted in an increase in caspase-3/7 activity and apoptosis of the injected cells. Almost all mice that received cells transfected with miR-200a developed tumors that failed to grow or that exhibited a marked reduction in size, indicating that miR-200a blunted the ability of meningioma cells to form tumors [186]. Tuchen et al. employed an orthotopic xenograft mouse model to assess the role of receptor tyrosine kinases (RTKs) in meningioma progression [187]. They used sorafenib and regorafenib RTK inhibitors, which targeted the phosphorylation of p44/42 ERK through the downregulation of the PDGFR. Monitoring tumor growth using small-animal MRI revealed that inhibition of RTKs inhibited growth and invasion of meningioma cells [187]. The availability of several mouse meningioma models represents a tool that can be exploited for further advances in meningioma biomarker discovery. Despite several limitations (Table 2), these pre-clinical models are continuously being optimized to enhance meningioma research. For example, the CRISPR-Cas 9 technology seems promising for next-generation mouse models of meningiomas. Moreover, it is relevant to develop new GEMMs that explore targetable somatic mutations found in human meningiomas such as TRAF7, AKT1, and PIK3CA, among others.

## 7. Conclusions

Meningiomas are the most prevalent primary intracranial tumors, accounting for 36% of all CNS tumors. There are various types and subtypes of meningiomas, bestowing them with a wide heterogeneity and complicating diagnosis. In addition, there are overlapping characteristics between benign and malignant subtypes, necessitating the presence of fast and effective diagnostic biomarkers.

Currently, there are no viable indicators of diagnosis, prognosis, or management of these tumors. Radiological imaging, mainly CT and MRI, is the main method of meningioma diagnosis. Unfortunately, imaging is not always suitable since it requires a big tumor size, presents overlapping findings with other CNS tumors, and needs continuous radiation exposure for follow-up. Imaging cannot anticipate the clinical behavior of meningiomas. All these limitations in current methods of diagnosis and prognosis necessitate the development of new meningioma biomarkers.

Previous WHO classification of meningiomas is based on histopathology; however, due to the heterogeneity of meningiomas, future diagnosis, prognosis, and therapy of meningiomas will likely be based on a multi-omics approach by combining genomic, proteomic, and epigenetic landscapes. The addition of CDKN2 A/B and pTERT mutations to the 2021 WHO classification to stratify Grade 3 meningiomas is a step in this direction. Other biomarkers are expected to follow suit to become an essential tool to guide therapy. Few of these biomarkers are being studied in clinical trials to develop targeted personalized therapies, including a phase II trial (NCT02523014) to investigate drugs in AKT1-mutant, SMO-mutant, or *NF2*-mutant meningiomas [188].

*NF2* gene mutations have been used as potential meningioma biomarkers, but proteomic-based biomarkers are better suited to accommodate meningioma diversity. Several prospective biomarkers are currently being researched such as serum protein expression patterns, CSF proteins, miRNAs, and lncRNAs. Furthermore, the use of the available meningioma animal models will facilitate the discovery of new tumor meningioma biomarkers. The ultimate diagnosis of meningiomas may require a panel of biomarkers of different types to cope with the heterogeneity of this disease. Such biomarkers when available will lead to fast and accurate stratification and grading of the different meningioma subtypes and enhance pre-operative and post-operative decision making. Importantly, these biomarkers may offer new targets for the development of new meningioma therapies including theranostic meningioma therapies. The majority of these molecular biomarkers are still experimental and need testing in clinical trials, but the addition of CDKN 2A/B and pTERT mutations into the 2021 WHO classification of meningiomas opens the door for the integration of other molecular biomarkers into diagnosis and the WHO grading system.

## Figures and Tables

**Table 1:** Measures of association of known biomarkers of meningiomas.

Biomarker Type	Known Biomarkers	Study Design	Clinical Use	Correlation with Grade	Description of Marker Usage and Its Effects	Reference and Year
Genomics	*NF2*, TRAF7, AKT1, SMO, and PIK3CA	Review	Diagnosis/Therapy	-	-	[29], 2020
	SMARCB1	Review	Diagnosis/Therapy	Grades 1 and 2	Genetic risk factor for sporadic multiple meningiomas	[29], 2020
	KLF4	Review	Diagnosis/Therapy	Grade 1	Downregulated in anaplastic meningiomas	[29], 2020; [65], 2017
	CDKN2A/B homozygous deletion	Cohort of 528 meningioma patients	Diagnostic/Prognostic	Grade 3 > Grade 2; absent in Grade 1	Faster progression to recurrence Higher mortality	[66], 2020
miRNA	miR-29c-3p and miR-219-5p	A study of 50 meningioma patients training set and 60 meningioma patients validation set compared to normal adjacent tissue	Diagnosis, Prognosis, and Therapy Response	Grades 1 > 2 > 3	Downregulation associated with advanced clinical stages of meningiomas and significant correlation with higher recurrence rates	[67], 2013
	miR-190a	A study of 50 meningioma patients training set and 60 meningioma patients validation set compared to normal adjacent tissue	Prognosis	Grades 1 < 2 < 3	Upregulation associated with advanced clinical stages of meningiomas, independent of other clinicopathological factors	[67], 2013
	miR-17-5p, miR-199a, miR-190a, miR-186-5p, miR-155-5p, miR-22-3p, miR-24-3p, miR-26b-5p, miR-27a-3p, miR-27b-3p, miR-96-5p, and miR-146a-5p	A study of 50 meningioma patients training set and 60 meningioma patients validation set compared to normal adjacent tissue	Diagnosis, Prognosis, Histological grade, and Radio-sensitivity	-	Significantly upregulated in meningioma samples	[67], 2013
	miR-219-5p, miR-106a-5p, miR-375, and miR-409-3p	20 pre-operative meningiomas and 20 healthy controls as discovery set Candidate miRNAs were validated individually in another 210 meningioma and 210 healthy controls	Non-invasive Diagnostic/Prognostic	miR-219-5p: Grades 3 > 2 > 1	Serum levels of the miRNA panel significantly increased in meningioma cases Serum levels of miR-219-5p positively correlated with higher meningioma grade	[68], 2016
	miR-197 and miR-224	20 pre-operative meningiomas and 20 healthy controls as discovery set Candidate miRNAs were validated individually in another 210 meningiomas and 210 healthy controls	Non-invasive Diagnostic/Prognostic	-	Serum levels significantly decreased in meningioma cases High serum miR-409-3p and low miR-224 expression significantly correlated with higher recurrence rates	[68], 2016
	Upregulation of miR-4286, miR-4695-5p, miR-6732-5p, miR-6855-5p, miR-7977, miR-6765-3p, and miR-6787-5p and downregulation of miR-1275, miR-30c-1-3p, miR-4449, miR-4539, miR-4684-3p, miR-6129, and miR-6891-5p	Study of 55 atypical meningioma patients (43 radio-sensitive and 12 radio-resistant meningiomas) and 6 arachnoid samples as control	Prognosis/response to radiotherapy	Grade 2	14 miRNAs significantly dysregulated in meningiomas Prediction of individual sensitivity to radiotherapy in patients resistant to radiotherapy Dysregulated miRNAs enriched in fatty acid biosynthesis and metabolism and TGFβ signaling pathways	[69], 2020
	miR-181d	Study collected meningioma tissues and plasma of 40 meningioma patients (16 Grade 1, 16 Grade 2, and 8 Grade 3 patients)	Non-invasive Diagnosis/Prognosis	Grades 1 < 2 < 3	Associated with tumor progression in plasma and tumor tissues	[70], 2021
LncRNA	LncRNA-LINC00460	A study of tissues from 32 meningioma patients and 5 normal control cases, in addition to in vitro studies in meningioma cell lines	Diagnosis	Grades 2 < 3	Upregulated in meningioma tissues and malignant cell lines	[71], 2020
	ISLR2, Lnc-GOLGA6A-1, AMH, and Grades 1 > 2	A study of 64 meningioma patients (with and without recurrence and of different WHO grades) that were subjected to RNA-seq; 90 samples validated using RT-qPCR	Prognosis and Pathogenesis	Lnc-MAST4-5: Grades 1 > 2, 3	ISLR2, Lnc-GOLGA6A-1, and AMH associated with recurrence risk	[72], 2022
	Lnc-00460	A study of 33 human meningioma tumor tissues and 10 normal meninges tissues, in addition to meningioma cell lines	Diagnosis	-	Upregulated in meningioma tissues and cell lines	[73], 2018
	LncRNA-NUP210, LncRNA-SPIRE2, LncRNA-SLC7A1, and LncRNA-DMTN	Review	Diagnosis/Prognosis	-	Upregulated in meningiomas Target microRNA-195	[74], 2023
Epigenetic	TIMP3, HOXA7, HOXA9, and HOXA10	Review	Prognosis	-	Hypermethylation associated with tumor progression and malignant transformation	[75], 2015; [76], 2020; [77], 2023
	TRAF7, KLF4, *NF2*, TRAKL, ARID1A, and AKT1	Retrospective analysis of formalin-fixed paraffin-embedded sections of 126 meningioma patients of different grades	Prognosis	-	Aberrant DNA methylation of these genes may be involved in the development and progression of meningiomas	[78], 2022; [77], 2023
	TIMP3, CDKN2A, and NDRG2	Review	Prognosis	-	Faster recurrence	[76], 2020; [77], 2023
	TP73, RSSF1A, and MAL2	Review	Prognosis	-	Hypermethylation increases risk of malignancy	[76], 2020; [77], 2023
	H3K27me3 histone modification	Retrospective study of 232 meningioma patients	Diagnosis/Prognosis	Grades 1 < 2 < 3	Loss of H3K27me3 methylation patterns correlated with high recurrence	[26], 2018
	Mutations in hTERT gene promoter	Study of 252 meningioma patients	Diagnosis/Prognosis	Grade 3 (aggressive)	Presence of hTERT promoter mutations means shorter time to progression	[79], 2016
	Mutations in hTERT gene promoter	Meta-analysis of 8 clinical trials	Diagnosis/Prognosis	Grades 1 < 2 < 3	Presence of hTERT promoter mutations resulted in higher recurrence rates and mortality This was a better prediction than WHO grading system	[80], 2019
Proteomic	APO-E and APO-J	Proteomic analysis of CSF from 4 meningioma patients and 4 patients with a non-brain	Diagnosis	Grade 2	Tumor progression marker	[81], 2012
	PTGDS	Clinical Study	Diagnosis	Grade 1	Associated with higher grade and early recurrence in intracranial meningiomas	[82], 2019
	Caspase-3, Amphiregulin, and VEFG-D	Screening cohort followed by a validation set of meningioma tissues and serum	Non-invasive diagnosis and prognosis	Grades 1 < 2, 3	The 3 proteins may constitute a panel that correlates with meningioma progression	[82], 2019
	EFEMP1	A study of CSF and serum of 45 meningioma patients and 30 healthy controls	Diagnosis		CSF and serum EFEMP1 levels significantly higher meningioma patients	[83], 2017
Histological	SSTR2A and Claudin-1	35 meningiomas, 10 intracranial schwannoma, and 10f hemangiopericytoma cases	Diagnosis	SSTRA: Grades 1, 2 > 3 Claudin-1: Grades 1, 2 < 3	Distinguishes meningiomas from schwannoma and hemangiopericytoma	[84], 2018
	CA9	Immunohistochemistry of paraffin-embedded sections of 25 Grade 1, 17 Grade 2, and 20 Grade 3 meningiomas	Prognosis	Grade 3	Associated with higher grade histology and common in recurrent tumors	[85], 2007
Metabolomic	Alanine and Glutamine/Glutamate	^1^H NMR of 23 Grade 1 and 10 Grade 2 meningioma tissues	Diagnosis/Prognosis	Glutamine metabolism: Grades 1 > 2	Predominantly elevated in Grade 2 meningiomas	[86], 2022
	Glycine/Serine	Validation of 43 meningioma patients	Diagnosis/Prognosis	Grades 1 > 2 > 3	Grade 1 associated with lower proliferation and longer progression-free survival	[87], 2021
	Choline/Tryptophan	Validation of 43 meningioma patients	Diagnosis/Prognosis	Grades 2, 3 > 1	Higher tryptophan/choline associated with shorter progression-free survival Similar incidence of Grades 1, 2, and 3	[87], 2021,
	Sphingolipid Galactosyl Ceramide	Discovery using LC-MS/MS and validation in 85 meningioma biopsies of different grades	Diagnosis/Prognosis	Grades 2, 3 > Grade 1	Higher levels in WHO Grades 2 and 3 than Grade 1	[88], 2023
	High acetate, threonine, N-acetyl-lysine, hydroxybutyrate, myoinositol, ascorbate, and total choline and low aspartate, glucose, isoleucine, valine, adenosine, arginine, and alanine	Metabolomics analysis using HRMAS NMR of 62 human meningioma samples	Diagnosis/Prognosis	Aggressive Grade 1 and Grade 2 have similar metabolic signature to Grade 3	Poor prognosis and high proliferation and histological grade	[89], 2020
Integrated systems of molecular/histological biomarkers	WHO grade, methylation class, and absence of chromosomes 1p, 6q, and 14q	Retrospective and prospective multi-center clinical study of 514 meningiomas and validation in 471 samples	Diagnosis/Prognosis		Nine-point scoring system Final scores of 3–5 → low risk of recurrence; 3–5 → intermediate risk; and score of 6–9 → high risk of recurrence	[90], 2021
	Mitotic index, *CDKN2A/B* homologous deletion, and alterations of copy number of specific chromosomes	Discovery cohort of 527 meningiomas and a validation set of 172 meningiomas	Diagnosis/Prognosis		Points scoring system Final scores of 0–1 → low risk of recurrence; 2–3 → intermediate risk; and score of 4 or more → high risk of recurrence	[91], 2022

**Table 2:** Advantages and limitations of xenograft and genetically engineered mouse models of meningiomas.

Mouse Model	Advantages	Limitations
Heterotopic xenograft model	Very reliable in terms of tumor take rates.	Lacks the key components of the meningioma’s specific microenvironment.
Orthotopic xenograft model	Very high tumor take rates for malignant meningiomas (100%). Immortalized benign meningioma cell lines produce tumor takes between 55% and 100%.	Tumor monitoring includes small-animal MRI, which is expensive and lacks ready availability. The need for an immunocompromised host *. Studies on interactions between tumor cells and the host immune system are not feasible *. The potent selective pressure during cell culture raises concerns that the utilized cells may not be representative of the original tumor *.
Genetically Engineered Mouse Models (GEMMs)	Accurately mimics human cancers with the presence of wild-type competitor cells modulating the cancer cells function. Mirrors human meningioma biology in terms of anatomy, histology, and genetic driver events. Facilitates the assessment of spatio-temporal susceptibility to meningioma tumorigenesis.	High financial costs for the generation and use of models. Time-consuming: may require several crosses and the time to tumor growth can be very long. The tumor take generally ranges from 30 to 80%. Unknown tumor growth rates and kinetics. Occasional production of non-meningeal tumors that induce early mortality in mice.

* These limitations are similar to the heterotopic xenograft model.
